# Roosting Ecology and the Evolution of Pelage Markings in Bats

**DOI:** 10.1371/journal.pone.0025845

**Published:** 2011-10-03

**Authors:** Sharlene E. Santana, Thomas O. Dial, Thomas P. Eiting, Michael E. Alfaro

**Affiliations:** 1 Center for Society and Genetics, University of California Los Angeles, Los Angeles, California, United States of America; 2 Department of Ecology and Evolutionary Biology, University of California Los Angeles, Los Angeles, California, United States of America; 3 Organismic and Evolutionary Biology, University of Massachusetts Amherst, Amherst, Massachusetts, United States of America; University of Western Ontario, Canada

## Abstract

Multiple lineages of bats have evolved striking facial and body pelage makings, including spots, stripes and countershading. Although researchers have hypothesized that these markings mainly evolved for crypsis, this idea has never been tested in a quantitative and comparative context. We present the first comparative study integrating data on roosting ecology (roost type and colony size) and pelage coloration patterns across bats, and explore the hypothesis that the evolution of bat pelage markings is associated with roosting ecologies that benefit from crypsis. We find that lineages that roost in the vegetation have evolved pelage markings, especially stripes and neck collars, which may function in crypsis through disruptive coloration and a type of countershading that might be unique to bats. We also demonstrate that lineages that live in larger colonies and are larger in size tend not to have pelage markings, possibly because of reduced predation pressures due to the predator dilution effect and a lower number of potential predators. Although social functions for pelage color patterns are also possible, our work provides strong support for the idea that roosting ecology has driven the evolution of pelage markings in bats.

## Introduction

**Figure 1:**
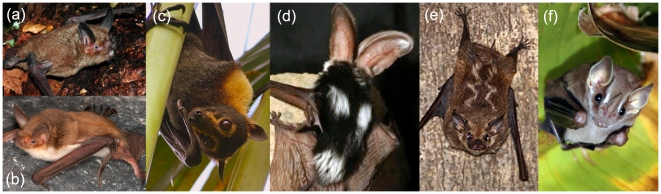
Main types of pelage markings seen across bat families, which were used as categories in this study. (a) uniform coloration, characterized by the absence of markings, shown in *Mystacina tuberculata* (Mystacinidae); (b) Countershading, shown in *Myotis vivesi* (Vespertilionidae); (c) Neck band, in *Pteropus conspicillatus* (Pteropodidae); (d) Spots, in the dorsum of *Euderma maculatum* (Vespertilionidae); and Stripes (e) in the dorsum of *Saccopteryx bilineata* (Emballonuridae) and (f) in the face of *Vampyressa pusilla* (Phyllostomidae), which is roosting under a leaf tent. Photo credits: Stuart Parsons (a), Marco Tschapka (b), U.S. National Park Service (c), and Jesús Molinari (e).

The evolution of mammalian coloration patterns has been linked to functions such as concealment from potential prey or predators, communication with con- and heterospecifics, and regulation of physiological processes such as thermoregulation and glare reduction (reviewed in [1]). Although bats are not renowned for their diversity in color, multiple lineages have evolved striking facial and body pelage makings, including spots, stripes and countershading (Figure 1). Researchers have long posed that these markings serve in crypsis through disruptive coloration or other forms of background matching, in particular for species that roost in open vegetation (reviewed in [2], [3]). To date, this hypothesis has never been tested in a quantitative and comparative context.

Bats generally occupy a nocturnal niche, and although they possess sight, most members of this order use vocal and olfactory rather than visual cues as a major means of perceiving their environment and communicating with conspecifics during social interactions [4], [5], [6], [7], [8], [9], [10], [11], [12]. Because of this potentially lower reliance on pelage markings for social communication, bats may represent an ideal model for studying the influence of environmental pressures on the evolution of pelage coloration. Previous studies of bat coloration have mostly been anecdotal or have focused on intraspecific or intrageneric variation for taxonomic purposes (compiled in [13]). Nevertheless, these studies have provided very detailed descriptions of the coloration of most bat species and have set the stage for comparative studies of the selective pressures shaping bat color patterns.

Much of the diversity in color patterns seen across mammals has evolved as part of their strategies to avoid visually-oriented predators [14], and this is also presumed for bat markings. While some mammals, such as skunks, have evolved markings that advertise their potential noxiousness or pugnacity (aposematism, [15], [16], [17]) or coloration patterns that deflect predatory attacks away from vulnerable areas of the body [1], many species have evolved coloration that makes them less conspicuous to predators. This strategy is part of crypsis [18], which comprises “all traits that reduce an animal's risk of becoming detected when it is potentially perceivable to an observer” [19]. For vision, crypsis includes features of the physical appearance (e.g. coloration) and behavioral traits to prevent detection (e.g. roost selection, activity period, etc.). Cryptic colorations can be achieved through mechanisms including background matching, disruptive coloration, and countershading [18], [20], [21], [22]. In background matching, an animal is less conspicuous due to an overall color or pattern that resembles the natural visual background of its environment (e.g., [23], [24], [25]). Concealment may also be achieved through pelage markings that create disruptive coloration. For example, some types of markings, such as spots and stripes, can visually break up the body's outline by creating patches that catch the eye of the observer and draw attention away from the body's shape [21], [26], [27]. Finally, mammals may be concealed by having a lighter ventral surface, which is thought to counteract the sun's effects when it shines on the animal's darker dorsal surface [28], [29], [30].

Bats inhabit not only a variety of naturally occurring roosts including caves, hollow trees, rock crevices, and foliage, but they also construct their own roosts, such as tents made from plant parts and cavities inside arboreal termite nests [31], [32]. Species living in these different roost types may not only differ in the visual environmental conditions to which they are exposed (e.g. luminance, color spectrum, etc.), but also in how salient they may appear to visually-oriented predators. Therefore, evolutionary pressures for crypsis and the color patterns through which it may be achieved can be expected to be different across roost types. In particular, camouflage towards visually-oriented predators might pose stronger selective pressures in species that roost in open vegetation, and disruptive coloration may be an important mechanism leading to crypsis in this type of roost. For example, the proboscis bat (*Rhynchonycteris naso*) often roosts on the surface of tree trunks [33]. This species presents dorsal stripes that likely serve in crypsis through disruptive coloration [2]. Similarly, many tent-making bats (families Phyllostomidae and Pteropodidae) have spots and stripes in their wings, facial and body pelage, and it is presumed that these also function in crypsis [2], [31].

Along with the type of roost used by bats, the number of individuals roosting together may also have an impact on the strategies used by these animals to avoid detection. Bat colony size spans six orders of magnitude, from solitary species to colonies of millions of individuals [13]. In many cases, bats may gain protection from predators by living in larger aggregations, in part due to the predator dilution effect, in which the risk of being eaten is lower for each individual bat as the colony becomes larger [34], [35]. This effect has been proposed as one of the reasons why larger bat colonies tend to emerge earlier than smaller colonies, even though the risk of detection by aerial predators is higher during that period [36]. Also, larger colonies tend to be formed by larger individuals (see below), thus the risk of mortality should be lower for species in larger colonies because they should have fewer predators than small-bodied species [37], [38].

Our goal is to integrate data on the pelage coloration and roosting ecology of bats to test the hypothesis that the evolution of pelage markings is associated with roosting ecologies that benefit from crypsis. We predict that species living in roosts that are exposed, in particular those in the vegetation, will have pelage markings such as countershading, neck bands, spots and stripes. In particular, we expect that the presence of spots and stripes will be strongly associated with roosting in the open. Pelage markings would function in crypsis through mechanisms including disruptive coloration and countershading. We also predict that pelage markings will be less prevalent in species living in larger colonies, because the *per capita* risk of predation should decrease with colony size due to predator dilution, and body size.

## Methods

### Pelage markings

Data on body markings were gathered from species' descriptions and photographs in Walker's Bats of the World [13] and Mammalian Species accounts (N = 914 species). When descriptions about coloration patterns in these sources were not clear, we used photographs from public scientific databases such as Animal Diversity Web (http://animaldiversity.ummz.umich.edu), Arkive (http://www.arkive.org), Bat Conservation International (http://www.batcon.org), and the Bat Conservation Trust (http://www.bats.org.uk) to corroborate the coloration pattern for particular species. Coloration of the face and body was scored for the presence or absence of each of the following markings: spots (circular areas of contrasting pelage), stripes (elongated areas of contrasting pelage), neck band (a band of contrasting and lighter pelage around the neck), and countershading (contrasting dorsoventral coloration) (Figure 1). When spots or stripes were present on the wings, these were also noted as markings since these are often visible during roosting. Uniform coloration was characterized by the absence of any of the color patterns described above. We created binary variables for each marking type by noting their presence (1) or absence (0) for each species. Geographic variation or sexual dimorphism for the presence of pelage markings was uncommon. We categorized species with this type of variation as presenting the pelage pattern (1) when this was predominant across populations.

### Roosting ecology

#### 1. Roost type

Data on the roost type used by species (n = 916) was gathered from Walker's Bats of the World [13] and reports compiled by the International Union for Conservation of Nature (IUCN, http://www.iucnredlist.org). Across all bats, we identified four main categories of roost type, which we used to classify species: (1) Exposed vegetation: species that roost on the surface of tree trunks or hanging from tree branches without any or minimal coverage from the foliage, (2) Concealed vegetation: species that roost inside or under shelters they have created in the vegetation, including tents made from leaves and cavities excavated inside arboreal termite nests, and species roosting under the leaf litter, (3) Caves, and (4) Crevices in rocks and cliffs. Since our goal was to explore whether bats have evolved markings in tandem with roosting in the vegetation and this habit seemed to integrate two roosting strategies (exposed and concealed vegetation), we pooled the data in three ways. First, we considered all species roosting in the vegetation together in one category (“All vegetation roosts” = categories 1 and 2 above) and those not roosting in the vegetation as another category (categories 3 and 4). Second, we considered species living in exposed vegetation roosts (category 1) separately and placed all the other species in another category (2–4 above). Third, we repeated the later procedure for species roosting in the concealed vegetation (category 2). Categorizing species in either type of vegetation roost allowed us to further explore if particular types of vegetation roosts were associated with the evolution of different pelage markings.

#### 2. Colony size and body mass

Colony size was recorded as the maximum reported aggregations in natural roosts of each species ([13], [35], [39], [40], [41], [42], [43], [44], [45], [46], [47], [48], [49], [50], [51]; n = 139, Table S1). For species in which colony size was reported as “a few hundred”, we rounded this value to 500. Similarly, we rounded colony size to 5,000 when the value was reported as “a few thousand”. Our dataset had fewer than 10 species in which colony size was reported as a few hundred or a few thousand. Body mass was taken from the literature [52] for all species for which we had colony size data. Colony size and body mass data were log_10_-transformed prior to analysis. Log-colony size and log-body mass were positively correlated under a phylogenetic context (Phylogenetic Generalized Least Squares regression, β = 1.434±0.338, t = 4.246, df = 136, P = 4.003 exp-05; Figure S1). Only log colony size was used in subsequent analyses since incorporating body mass yielded redundant results.

### Phylogenetic analyses

We conducted phylogenetic logistic regressions to evaluate if the evolution of pelage markings (presence/absence of spots, stripes, neck collar, countershading) was related to roost type (categorical variable) and log colony size (continuous variable). These regressions were run using a pruned version of the Jones et al. supertree of bats (916 species; [53], [54]) and the PLoGReg Matlab (Mathworks, Natick, MA, USA) function written by Ives and Garland [55]. This function simultaneously tests for phylogenetic signal while conducting regressions. Our phylogenetic logistic regression models included a binary dependent variable (marking presence) and the two independent variables (roost type, log colony size) simultaneously. A bootstrapping procedure involving 1,000 simulations was used to generate the confidence intervals and test for statistical significance of the slope and intercept of the regression models. Convergence of model parameters was achieved in all cases after these simulations.

## Results

**Figure 2:**
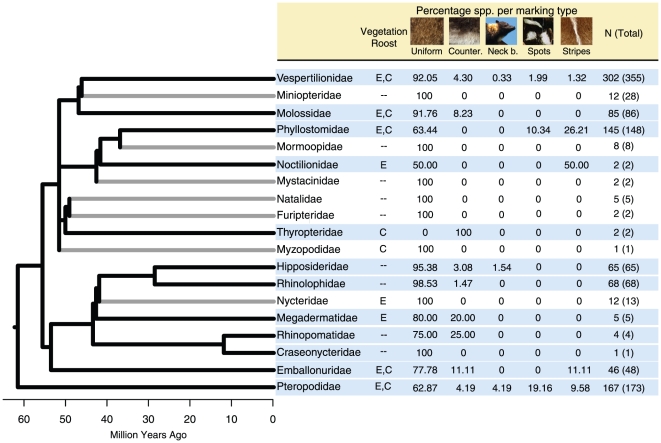
Bat families included in this study, their evolutionary relationships, roosting ecology, percentage of species with each type of pelage marking (uniform, count. = countershading, neck b. = neck band, spots, and stripes), and sample sizes. Tree branches for families that have pelage markings are colored in black, and the names and data for these are also highlighted in blue. For vegetation roost, E: exposed vegetation, and C: concealed vegetation. Sample sizes are given in number of species per family. The total number of species per family are given in parentheses (following [13], [53], [68]).

Uniform coloration is the most predominant pelage coloration type across bat species (Figure 2). However, pelage markings are present in species from 12 out of the 19 families studied. While some clades with low or intermediate species richness levels are characterized by only one type of marking (e.g. Molossidae, Thyropteridae, Rhinolophidae), other species-rich clades have evolved all the types of markings considered in this study (e.g., Vespertilioniade, Phyllostomidae, Pteropodidae). Out of the marking types, countershading coloration was the most prevalent across lineages, followed by stripes, neck bands and spots.

### Evolution of pelage markings and roosting ecology

Phylogenetic signal was found to be high and statistically significant in all the variables tested. The evolution of body markings was significantly associated with roosting in both exposed and concealed vegetation (Table 1). In all cases, the presence of pelage markings was positively related to using vegetation roosts (β_All_ = 1.415, β_exposed_ = 1.306, β_concealed_ = 1.349; P<0.05, see table for confidence intervals). The presence of markings, pooled together, was negatively related to colony size, although this relationship was not significant (P>0.1).

**Table 1:** Results from phylogenetic logistic regressions relating the presence of pelage markings and roosting ecology.

Marking type	Predictor	All vegetation roosts		Roosts in exposed vegetation		Roosts in concealed vegetation	
		β	*P*	β	*P*	β	*P*
**All markings**	Roost	(0.619, 1.415, 2.163)	<0.0001	(0.448, 1.306, 2.128)	<0.0001	(0.267, 1.349, 2.784)	0.04
	Colony size	(−0.451, −0.158, 0.118)	0.26	(−0.487, −0.173, 0.124)	0.28	(−0.369, −0.112, 0.159)	0.40
**Spots**	Roost	(−0.957, 0.024, 1.969)	0.85	(−0.385, 0.379, 1.272)	0.28571	(−2.082, −0.880, −0.007)	0.08
	Colony size	(−1.852, −1.827, −0.091)	0.06	(−1.873, −0.412, −0.156)	0.04	(−1.675, −0.483, −0.192)	0.02
**Stripes**	Roost	(0.563, 1.361, 4.288)	0.02	(0.050, 0.468, 1.941)	0.08	(−1.252, 1.085, 3.636)	0.28
	Colony size	(−0.524, −0.180, −0.087)	0.04	(−0.646, −0.237, −0.015)	0.10	(−1.711, −0.369, 0.197)	0.14
**Neck band**	Roost	(0.569, 2.137, 4.994)	<0.0001	(0.568, 1.207, 2.721)	<0.0001	(1.154, 1.265, 2.357)	0.02
	Colony size	(0.177, 0.464, 0.796)	<0.0001	(0.165, 0.356, 0.596)	<0.0001	(0.791, 0.964, 1.222)	<0.0001
**Countershading**	Roost	(−0.432, 0.319, 0.825)	0.50	(−0.576, 0.228, 1.039)	0.46	(−1.266, 0.321, 1.590)	0.60
	Colony size	(−0.299, −0.056, 0.189)	0.76	(−0.327, −0.025, 0.148)	0.72	(−0.449, −0.078, 0.212)	0.58

Results are given for markings and vegetation roosts pooled together (“All markings” and “All vegetation roosts”) as well as separately. Slopes presented (β) are bootstrapped bounds of confidence intervals (lower bound, mean, upper bound) and their associated P-value.

When markings were examined separately, the evolution of spots was not associated with roosting in the vegetation (P>0.1), but there was a significant effect of colony size in this variable (β_All_ = −1.827, β_exposed_ = −0.412, β_concealed_ = −0.483; P<0.05). Species living in larger colonies were less likely to have spots. Similarly, species living in large groups tended to lack stripes (β_All_ = −0.180, P = 0.04), and this marking type was also associated with roosting in the vegetation (β_All_ = 1.361, P = 0.02). The evolution of neck bands was associated with both roosting in all types of vegetation and with colony size (for roost: β_All_ = 2.137, β_exposed_ = 1.207, β_concealed_ = 1.265; P<0.05). Species living in larger colonies tended to have neck bands and roost in the vegetation. The presence of countershading was not associated with the variables describing roosting ecology examined here (P>0.1).

## Discussion

To our knowledge, this is the first broad comparative study revealing an evolutionary association between roosting ecology and pelage markings in bats. Despite the wide diversity of environments inhabited by these mammals, we found that the presence of pelage markings is positively associated with roosting in the vegetation. This finding supports our predictions and suggests that pelage markings function in crypsis and may constitute an important adaptation to avoid predation. We found that roosting in the vegetation is associated with the evolution of stripes and neck bands. The function of stripes as a camouflage strategy in a vegetation background has been documented in a wide array of animals, spanning insects [56], [57], fish [58], and mammals [59], [60]. Experimental evidence in these systems further supports the idea that disruptive coloration of prey, such as that caused by stripes, lowers detection by visually-oriented predators [21]. Therefore, it is likely that stripes function in crypsis in bats. According to Cott [22], the visual recognition of objects is mainly enabled by the continuity in the object's surface and its bounding by a specific contour or outline. When an animal has stripes, its overall shape appears to be subdivided into separate objects that are harder to integrate as the original shape. This effect might be more pronounced when the stripes seem to touch the outline of the animal and blend into the background, and when they provide a sharp contrast within the fur [21]. Many of the bats that present stripes are tent-making species, meaning that they modify leaves, stems and other plant parts to make a shelter [3]. These species often present contrasting white facial stripes (Figure 1f; family Stenodermatinae) that could contribute to a cryptic appearance when seen from below [2], [31]. We found some support for this idea in our dataset, but sample sizes for tent-making bats with facial stripes is relatively small to yield significant results (β_tent roost_ = 2.582, P>0.1, results not shown). In general, crypsis could not only be achieved through disruptive coloration caused by facial and body stripes, but also by blending with the patterns of light and shadows that are caused by the sunlight peeking through small gaps in the leaf tents.

Countershading patterns, characterized by a darker dorsal surface and a lighter ventral surface, are strongly related to postural behaviors in some mammals. In primates, countershading is strongly present in species of any size that frequently use horizontal locomotion positions, possibly because fitness benefits are gained from increased crypsis during these behaviors [29]. Neck bands may be a form of countershading that is particular to bats that roost in the vegetation [3]. Indeed, we found that neck bands are present more commonly in species that roost in the open (Table 1). Like most other bats, the species in which neck bands are present (Vespertilionidae, Hipposideridae and Pteropodidae) roost upside down almost exclusively, thus these bats may achieve increased crypsis by having lighter colors in the anterior portion of the body and darker colors in the posterior part of the body. If a uniformly-colored bat roosting in the open vegetation were exposed to the sun from above, it would exhibit a lighter posterior surface and produce a self-cast shadow, creating a gradient in its coloration. A neck band would provide a lighter anterior coloration that would reduce the light gradient across the animal's body, thereby appearing more two-dimensional and less conspicuous when viewed [28], [61]. Prey items that present countershading are harder to detect by birds [62], which are among the chief natural predators of bats. Interestingly, the evolution of dorsoventral countershading was not related to roosting in the vegetation or any other roost type, thus it remains unclear if or how this coloration pattern may function in crypsis. Furthermore, since natural predators of bats (birds of prey, snakes, carnivorans, [46]) span a range of sensory modalities for prey detection, different strategies for crypsis may evolve depending on which predators are most important for any given lineage. For example, pressures for cryptic coloration may not be as high in species whose predators rely mostly on olfaction to find prey. More detailed information on bat roosting ecology and behavior, and the type and abundance of their predators would further expand our understanding of the ecological mechanisms driving the evolution of pelage patterns.

Being larger and living in larger groups is associated with lower individual predation risk for many mammals [34], [35], [37], [38]. For all marking types except for neck collars, we found support for our prediction that the presence of pelage markings would decrease with colony size and body mass. Along with roost type, we considered these variables as proxies for predation risk. We recognize at least three alternative explanations for the decrease in pelage markings in bats that are larger and live in more numerous aggregations. First, as predicted by theory, living in larger colonies and being larger may lower the risk of predation, so there would be less pressure for the evolution of pelage markings in these species. Second, roosts where spots and stripes are advantageous for crypsis may be able to house only small groups of individuals (e.g. tents in the vegetation, plant surfaces), so pressures for small group and body size would parallel pressures for the presence of these markings. This would also explain the opposite trend observed in species with neck collars, which are often large and live in large groups in open vegetation and would not have the spatial constraint described above. Finally, an inverse relationship between the presence of markings and colony size might be due to other, social or ecological factors not measured here, including social communication.

Two caveats are in order. First, pelage color patterns across mammals also serve as signals to conspecifics during social interactions; and these may serve to identify individuals, assess condition, highlight behaviors, and other social functions [e.g.], [ 63], [64], [65,66]. This social role of pelage markings complicates the study of their function in concealment. The importance of bat pelage markings as cues during social interactions is poorly known, thus explanations relating sociality to the evolution of some of these traits cannot be excluded until more data become available. In particular, data describing the relationship among colony size, social and mating systems, would allow elucidating specific mechanisms that connect the evolution of pelage markings with colony size. This may be particularly relevant for the case of neck bands, stripes and spots, all of which are significantly related to colony size and could act as visual cues during social interactions. For example, some spotted pelage is the result of eversible epaulettes that are used in mating displays by some male pteropodid bats [67], which is also a group that presents a high reliance on vision. It is unclear if the importance of spots during visual mating displays is widespread across bats, since most of the species with this trait lack sexual dimorphism [13]. Moreover, it is also unknown if these markings originally evolved to function in crypsis and were later co-opted as social cues. Second, we found that colony size is correlated with body mass, and thus the statistical effect of these two variables is hard to disentangle within an evolutionary context. However, for the purposes of our study, the positive association between colony size and body mass does not pose conflicts to test our predictions, since both larger colonies and larger animals would be expected to be under lower predation risk and thus have lower pressures for crypsis.

Bats are one of the most ecologically and morphologically diverse groups of mammals, and present a unique system within which to investigate evolutionary correlates of pelage coloration. We integrate morphological and ecological data across hundreds of bat species to investigate how coloration patterns may be related to roosting ecology. Our study supports the idea that bat pelage markings have evolved in tandem with roosting in the vegetation and colony size, with these being proxies for predation risk. We illustrate how different types of markings may evolve under different ecological conditions, with stripes and neck bands being especially associated with vegetation roosts. Our work provides the basis for future experimental studies testing the salience of pelage markings under specific roosting conditions, and will serve to further the understanding of bat ecology, life history and evolution.

## Supporting Information

Figure S1 **Relationship between phylogenetically-adjusted body mass and maximum colony size (PIC = Phylogenetic Independent Contrasts).** Regression parameters from Phylogenetic Generalized Least Squares regression: β = 1.434±0.338, t = 4.246, df = 136, P = 4.003 exp-05, n = 139.
(PDF)

Table S1 **Maximum colony size and bibliographic sources for the species in our dataset.**
(PDF)
