# Supplementary material for: Roosting Ecology and the Evolution of Pelage Markings in Bats
Source: PLoS One. 2011 Oct 3;6(10):e25845. doi: 10.1371/journal.pone.0025845 (PMC3185059; doi:10.1371/journal.pone.0025845)

Figure S1: Relationship between phylogenetically-adjusted body mass and maximum colony size (PIC = Phylogenetic Independent Contrasts). Regression parameters from Phylogenetic Generalized Least Squares regression: $\beta = 1.434 \pm 0.338$, $t = 4.246$, df = 136, $P = 4.003$ exp-05, $n = 139$.

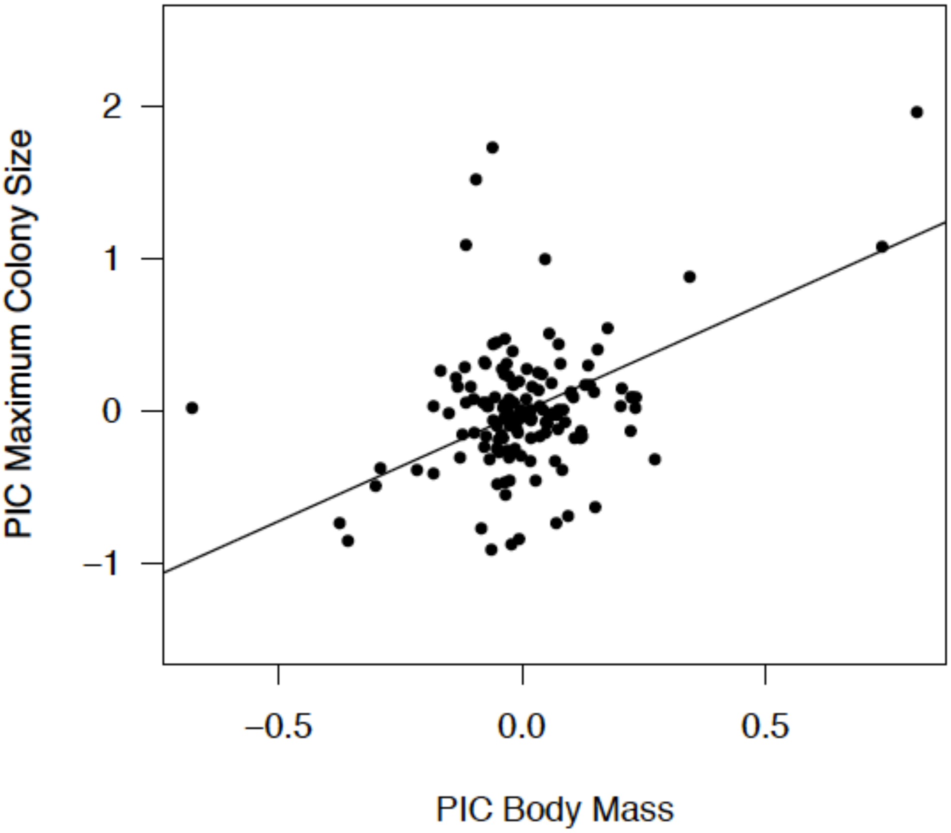

Supplement: Figure S1 — Relationship between phylogenetically-adjusted body mass and maximum colony size (PIC = Phylogenetic Independent Contrasts). Regression parameters from Phylogenetic Generalized Least Squares regression: β = 1.434±0.338, t = 4.246, df = 136, P = 4.003 exp-05, n = 139. (PDF) [file pone.0025845.s001.pdf]
