# Supplementary material for: Roosting Ecology and the Evolution of Pelage Markings in Bats
Source: PLoS One. 2011 Oct 3;6(10):e25845. doi: 10.1371/journal.pone.0025845 (PMC3185059; doi:10.1371/journal.pone.0025845)
Supplement: Table S1 — Maximum colony size and bibliographic sources for the species in our dataset. (PDF) [file pone.0025845.s002.pdf]

Table S1: Maximum colony size and bibliographic sources, listed below, for the species in our dataset

Family	Species	Max. Colony Size	Source
Craseonycteridae	*Craseonycteris thonglongyai*	500	10
Emballonuridae	*Coleura afra*	50000	4
Emballonuridae	*Cyttarops alecto*	10	14
Emballonuridae	*Emballonura monticola*	150	4
Emballonuridae	*Emballonura raffrayana*	30	2
Emballonuridae	*Emballonura semicaudata*	100	10
Emballonuridae	*Peropteryx macrotis*	80	14
Emballonuridae	*Rhynchonycteris naso*	45	14
Emballonuridae	*Saccopteryx bilineata*	50	14
Emballonuridae	*Saccopteryx leptura*	9	14
Emballonuridae	*Taphozous australis*	10	2
Emballonuridae	*Taphozous mauritianus*	30	13
Emballonuridae	*Taphozous melanopogon*	4000	4
Emballonuridae	*Taphozous theobaldi*	1000	4
Furipteridae	*Furipterus horrens*	250	4
Hipposideridae	*Aselliscus tricuspidatus*	100	4
Hipposideridae	*Hipposideros armiger*	200	5
Hipposideridae	*Hipposideros ater*	100	2
Hipposideridae	*Hipposideros caffer*	500000	4
Hipposideridae	*Hipposideros calcaratus*	1000	2
Hipposideridae	*Hipposideros cervinus*	300000	2
Hipposideridae	*Hipposideros commersoni*	100000	13
Hipposideridae	*Hipposideros diadema*	8000	2

Family	Species	Max. Colony Size	Source
Hipposideridae	*Hipposideros fulvus*	200	5
Hipposideridae	*Hipposideros lankadiva*	7000	5
Hipposideridae	*Hipposideros maggietaylorae*	70	2
Hipposideridae	*Hipposideros speoris*	1000	5
Hipposideridae	*Hipposideros turpis*	5000	10
Hipposideridae	*Rhinonicteris aurantia*	5000	4
Hipposideridae	*Triaenops persicus*	100	13
Megadermatidae	*Cardioderma cor*	80	7
Megadermatidae	*Lavia frons*	2	9
Megadermatidae	*Macroderma gigas*	800	7
Megadermatidae	*Megaderma lyra*	2000	7
Megadermatidae	*Megaderma spasma*	30	7
Miniopteridae	*Miniopterus australis*	100000	2
Miniopteridae	*Miniopterus medius*	10000	2
Miniopteridae	*Miniopterus natalensis*	110000	4
Molossidae	*Chaerephon jobensis*	350	2
Molossidae	*Chaerephon plicatus*	500	5
Molossidae	*Chaerephon pumilus*	2500	13
Molossidae	*Cheiromeles torquatus*	200000	10
Molossidae	*Cynomops greenhalli*	75	4
Molossidae	*Cynomops planirostris*	100	14
Molossidae	*Eumops auripendulus*	20	4
Molossidae	*Eumops glaucinus*	20	4
Molossidae	*Molossus rufus*	500	4
Molossidae	*Molossus sinaloae*	76	14
Molossidae	*Mops condylurus*	1000	13

Family	Species	Max. Colony Size	Source
Molossidae	*Mormopterus beccarii*	50	2
Molossidae	*Otomops martiensseni*	200	11
Molossidae	*Tadarida aegyptiaca*	600	5
Mormoopidae	*Mormoops megalophylla*	500000	4
Noctilionidae	*Noctilio leporinus*	100	14
Nycteridae	*Nycteris grandis*	6	13
Nycteridae	*Nycteris hispida*	20	4
Nycteridae	*Nycteris thebaica*	1000	4
Phyllostomidae	*Anoura geoffroyi*	88	4
Phyllostomidae	*Artibeus jamaicensis*	20	9
Phyllostomidae	*Artibeus lituratus*	20	14
Phyllostomidae	*Brachyphylla cavernarum*	10000	4
Phyllostomidae	*Carollia perspicillata*	1000	14
Phyllostomidae	*Choeroniscus minor*	8	4
Phyllostomidae	*Desmodus rotundus*	2000	4
Phyllostomidae	*Diphylla ecaudata*	500	14
Phyllostomidae	*Glossophaga soricina*	50	9
Phyllostomidae	*Lampronycteris brachyotis*	300	4
Phyllostomidae	*Leptonycteris curasoae*	1000	14
Phyllostomidae	*Leptonycteris nivalis*	10650	10
Phyllostomidae	*Lophostoma silvicolum*	10	4
Phyllostomidae	*Macrophyllum macrophyllum*	59	4
Phyllostomidae	*Mesophylla macconnelli*	8	4
Phyllostomidae	*Micronycteris megalotis*	20	4
Phyllostomidae	*Micronycteris minuta*	20	4
Phyllostomidae	*Micronycteris schmidtorum*	20	4

Family	Species	Max. Colony Size	Source
Phyllostomidae	*Phyllostomus discolor*	400	14
Phyllostomidae	*Phyllostomus elongatus*	15	4
Phyllostomidae	*Phyllostomus hastatus*	100	4
Phyllostomidae	*Platyrrhinus lineatus*	10	4
Phyllostomidae	*Trachops cirrhosus*	6	4
Phyllostomidae	*Uroderma bilobatum*	59	14
Phyllostomidae	*Vampyressa pusilla*	5	14
Phyllostomidae	*Vampyrodes caraccioli*	4	14
Phyllostomidae	*Vampyrum spectrum*	5	14
Pteropodidae	*Balionycteris maculata*	14	3
Pteropodidae	*Casinycteris argynnis*	1	4
Pteropodidae	*Chironax melanocephalus*	8	4
Pteropodidae	*Cynopterus brachyotis*	12	4
Pteropodidae	*Cynopterus horsfieldii*	12	4
Pteropodidae	*Cynopterus sphinx*	25	5
Pteropodidae	*Dobsonia praedatrix*	12	2
Pteropodidae	*Eidolon helvum*	1000000	6
Pteropodidae	*Eonycteris spelaea*	4000	5
Pteropodidae	*Epomophorus labiatus*	70	6
Pteropodidae	*Epomops franqueti*	3	4
Pteropodidae	*Hypsignathus monstrosus*	25	7
Pteropodidae	*Macroglossus minimus*	3	6
Pteropodidae	*Macroglossus sobrinus*	10	1
Pteropodidae	*Megaerops ecaudatus*	1	1
Pteropodidae	*Micropteropus pusillus*	2	6
Pteropodidae	*Myonycteris torquata*	1	1

Family	Species	Max. Colony Size	Source
Pteropodidae	*Notopteris macdonaldi*	300	4
Pteropodidae	*Nyctimene albiventer*	2	2
Pteropodidae	*Paranyctimene raptor*	4	1
Pteropodidae	*Pteropus alecto*	100000	6
Pteropodidae	*Pteropus conspicillatus*	52200	8
Pteropodidae	*Pteropus hypomelanus*	5000	2
Pteropodidae	*Pteropus neohibernicus*	1000	1
Pteropodidae	*Pteropus poliocephalus*	220000	6
Pteropodidae	*Pteropus samoensis*	40	1
Pteropodidae	*Pteropus scapulatus*	100000	4
Pteropodidae	*Pteropus tonganus*	7000	6
Pteropodidae	*Pteropus vampyrus*	150000	4
Pteropodidae	*Rousettus aegyptiacus*	9000	6
Pteropodidae	*Rousettus amplexicaudatus*	800	6
Pteropodidae	*Scotonycteris zenkeri*	1	4
Pteropodidae	*Syconycteris australis*	1	2
Rhinolophidae	*Rhinolophus cornutus*	100	12
Rhinolophidae	*Rhinolophus hipposideros*	50	9
Rhinolophidae	*Rhinolophus landeri*	1000	12
Rhinolophidae	*Rhinolophus lepidus*	400	4
Rhinolophidae	*Rhinolophus luctus*	2	12
Rhinolophidae	*Rhinolophus malayanus*	3000	12
Rhinolophidae	*Rhinolophus megaphyllus*	1000	2
Rhinolophidae	*Rhinolophus paradoxolophus*	50	12
Rhinolophidae	*Rhinolophus pearsonii*	35	12
Rhinolophidae	*Rhinolophus pusillus*	1500	12

Family	Species	Max. Colony Size	Source
Rhinopomatidae	*Rhinopoma hardwickii*	1050	11
Rhinopomatidae	*Rhinopoma microphyllum*	1000	4
Vespertilionidae	*Chalinolobus gouldii*	50	4
Vespertilionidae	*Chalinolobus morio*	150	11
Vespertilionidae	*Eptesicus fuscus*	700	4
Vespertilionidae	*Glauconycteris humeralis*	30	4
Vespertilionidae	*Glauconycteris poensis*	30	4
Vespertilionidae	*Ia io*	55	5
Vespertilionidae	*Kerivoula papillosa*	6	4
Vespertilionidae	*Kerivoula pellucida*	6	4
Vespertilionidae	*Kerivoula phalaena*	6	4
Vespertilionidae	*Lasiurus borealis*	100	4
Vespertilionidae	*Murina cyclotis*	3	5
Vespertilionidae	*Myotis bechsteinii*	15	9
Vespertilionidae	*Myotis bocagii*	1	13
Vespertilionidae	*Myotis dasycneme*	600	10
Vespertilionidae	*Myotis myotis*	500	9
Vespertilionidae	*Myotis nattereri*	30	9
Vespertilionidae	*Myotis nigricans*	1000	14
Vespertilionidae	*Neoromicia nanus*	150	4
Vespertilionidae	*Nyctalus noctula*	500	11
Vespertilionidae	*Nyctophilus geoffroyi*	100	4
Vespertilionidae	*Nyctophilus timoriensis*	2	2
Vespertilionidae	*Perimyotis subflavus*	10	9
Vespertilionidae	*Pipistrellus ceylonicus*	200	5
Vespertilionidae	*Pipistrellus tenuis*	20	5

Family	Species	Max. Colony Size	Source
Vespertilionidae	*Scotophilus dinganii*	12	13
Vespertilionidae	*Scotophilus heathii*	50	5
Vespertilionidae	*Scotophilus kuhlii*	300	5
Vespertilionidae	*Scotorepens sanborni*	100	2
Vespertilionidae	*Tylonycteris pachypus*	40	4
Vespertilionidae	*Tylonycteris robustula*	40	4
Vespertilionidae	*Vespadelus pumilus*	54	15

Maximum colony size references

1. Mickleburgh SP, Hutson AM, Racey PA (1992) Old World fruit bats: an action plan for their conservation. Gland, Switzerland: IUCN.

2. Bonaccorso FJ, Reid FA, International C (1998) Bats of Papua New Guinea. Washington DC: Conservation International.

3. Hodgkison R, Balding ST, Akbar Z, Kunz TH (2003) Roosting ecology and social organization of the spotted-winged fruit bat, *Balionycteris maculata* (Chiroptera: Pteropodidae), in a Malaysian lowland dipterocarp forest. J Trop Ecol 19: 667-676.

4. Nowak RM, Walker EP (1994) Walker's bats of the world. Baltimore: Johns Hopkins University Press.

5. Bates P, Harrison D (1998) Bats of the Indian Subcontinent. Kent, England: Harrison Zoological Museum.

6. Hosken D (1998) Testes mass in megachiropteran bats varies in accordance with sperm competition theory. Behav Ecol Sociobiol 44: 169-177.

7. Langevin P, Barclay RMR (1990) *Hypsignathus monstrosus*. Mammalian Species 357: 1-4.

8. Garnett S, Whybird O, Spencer H (1999) The conservation status of the spectacled flying fox *Pteropus conspicillatus* in Australia. Aust Zool 31: 38-54.

9. Wilkinson GS, South JM (2002) Life history, ecology and longevity in bats. Aging Cell 1: 124-131.
10. Hutson AM (2001) Microchiropteran bats: global status survey and conservation action plan. Gland, Switzerland: World Conservation Union.
11. Hosken D (1997) Sperm competition in bats. Proc Roy Soc B 264: 385-392.
12. Csorba GS, Ujhelyi P, Thomas N (2003) Horseshoe bats of the world (Chiroptera: Rhinolophidae). Shropshire, UK: Alana Books.
13. Taylor PJ (2000) Bats of southern Africa. Scottsville, South Africa: University of Natal Press.
14. Reid F (2009) Field guide to the mammals of Central America and Southeast Mexico. New York: Oxford University Press.
15. Law BS, Anderson J (2000) Roost preferences and foraging ranges of the eastern forest bat *Vespadelus pumilus* under two disturbance histories in northern New South Wales, Australia. Aust Ecol 25: 352-367.
